# Measurement properties of Chinese-version assessment tools of self-efficacy in patients with rheumatoid arthritis: a COSMIN-based systematic review

**DOI:** 10.1186/s41687-026-01135-9

**Published:** 2026-07-07

**Authors:** Linfei Ding, Qingli Jiang, Yan Zhang, Xiaoyu Li

**Affiliations:** 1 Mianyang Central Hospital, School of Medicine, University of Electronic Science and Technology of China, Mianyang, China; 2 University of Electronic Science and Technology of China, Chengdu, China

**Keywords:** Rheumatoid arthritis, Self-efficacy, Assessment tools, Measurement properties, Systematic review

## Abstract

**Purpose:**

This study aimed to systematically evaluate the measurement properties and methodological quality of self-efficacy assessment tools for rheumatoid arthritis(RA) patients in China, to provide evidence-based recommendations for clinicians and researchers in selecting appropriate tools.

**Methods:**

A comprehensive literature search was conducted in the following databases: CNKI, VIP, Wanfang, SinoMed, PubMed, Embase, Web of Science, Scopus, and CINAHL, to identify studies assessing the measurement properties of Chinese versions of the assessment tools of self-efficacy in patients with RA. The search covered all publications up to April 2025. The reported measurement properties included content validity, structural validity, internal consistency, reliability, and hypotheses testing for construct validity. Studies were eligible if: (1) The study population included patients with RA; (2) was the development, cross-cultural adaptation or validation of a self-efficacy assessment tool for RA; (3) evaluated at least one measurement property of a self-efficacy assessment tool for RA.; (4) The study was published in a peer-reviewed journal; (5) The publication language was English or Chinese. Studies were excluded if: (1) The tool was designed to measure only a single dimension of self-efficacy (e.g., medication adherence, exercise self-efficacy); (2) Studies involving non-Chinese populations; (3) The study was a secondary research article (e.g., review, systematic review, conference abstract); (4) The full text of the article was not available. Two reviewers independently screened the literature and extracted data. The measurement properties and methodological quality of the included tools were assessed using the Consensus-based Standards for the Selection of Health Measurement Instruments (COSMIN), and final recommendations were formulated accordingly.

**Results:**

A total of seven studies were included, covering seven Chinese versions of the assessment tools of self-efficacy in patients with RA. Among them, the Chinese version of 8-item Arthritis Self-Efficacy Scale (C-ASES-8) demonstrated “sufficient” content validity, with moderate-quality evidence supporting “sufficient” internal consistency. It was categorized as “A” and is recommended for use. The Chinese version of Joint Protection Self-Efficacy Scale (C-JP-SES) (two-factor structure) showed high-quality evidence indicating insufficient structural validity and was categorized as “C,” therefore not recommended. The Chinese versions of JP-SES (single-factor structure), ASES, and Rheumatoid Arthritis Self-Efficacy Scale (RASE) did not meet the criteria for either “A” or “C” and were categorized as “B,” indicating they may be temporarily recommended pending further validation.

**Conclusion:**

The Chinese-version assessment tools of self-efficacy in patients with RA are diverse. Although the C-ASES-8 has met the recommended standards for use, comprehensive evaluations of its psychometric properties remain insufficient. Future research should aim to thoroughly validate all Chinese versions of the rheumatoid arthritis self-efficacy scales (including the C-ASES-8) and provide complete reports on their psychometric properties, to offer more reliable evidence to support instrument selection in clinical practice and research.

**Supplementary Information:**

The online version contains supplementary material available at https://doi.org/10.1186/s41687-026-01135-9.

## Introduction

Rheumatoid arthritis (RA) is a chronic autoimmune disease characterized primarily by synovial inflammation [1]. As the disease progresses, it can lead to irreversible joint damage and functional impairment [1]. Patients with RA often experience symptoms such as joint pain, stiffness, swelling, restricted mobility, and systemic fatigue, which severely affect their ability to perform daily and occupational tasks [2]. These symptoms negatively impact both physical and mental health, reduce quality of life [3], and impose a substantial economic and social burden [4]. In China, the prevalence of RA is approximately 0.36% to 0.5%, with an upward trend observed in recent years [5]. Given the absence of a definitive cure, strengthening comprehensive patient management—particularly by enhancing self-management capacity—has become increasingly important.

Self-efficacy refers to an individual’s confidence in their ability to perform the behaviors required to achieve specific goals; its level directly influences the effort and persistence exerted during goal attainment [6, 7]. Studies have demonstrated that higher levels of self-efficacy enable RA patients to achieve better disease self-management, improve daily functioning and pain control [8], enhance quality of life, and are strongly associated with positive outcomes such as improved emotional well-being and greater medication adherence [9]. Therefore, assessing self-efficacy levels before and after interventions is essential for the development and evaluation of individualized management strategies in RA care.

Self-efficacy assessment tools for RA patients are primarily based on self-report measures, commonly categorized as patient-reported outcome measures (PROMs) [10]. Several instruments have been developed for this purpose—including the Joint Protection Self-Efficacy Scale (JP-SES), the Arthritis Self-Efficacy Scale (ASES), the 8-item Arthritis Self-Efficacy Scale (ASES-8), and the Rheumatoid Arthritis Self-Efficacy Scale (RASE), along with their Chinese adaptations—yet significant gaps remain in understanding their comparative psychometric performance. A recent COSMIN-based systematic review synthesized global evidence on these instruments in patients with RA [11]. However, its broad inclusion of diverse populations may limit the contextual applicability of its findings. Moreover, among Chinese patients with RA, these instruments have largely been applied in isolation, with few head-to-head comparisons and no comprehensive evaluation of their psychometric properties or supporting evidence. Therefore, a systematic review is warranted to critically appraise, compare, and synthesize the psychometric evidence of these instruments in Chinese RA populations.

This study conducted a systematic evaluation of the psychometric properties and methodological quality of Chinese versions of RA self-efficacy assessment tools based on the Consensus-based Standards for the Selection of Health Measurement Instruments (COSMIN) [12]. By focusing exclusively on Chinese populations and culturally adapted instruments, this review addresses a granularity of evidence that global syntheses cannot provide. The aim is to provide evidence-based guidance for researchers and clinicians in selecting appropriate instruments in the Chinese context, enhance the evaluation effectiveness of these tools locally, and lay the foundation for scientifically assessing the impact of clinical interventions on RA patients’ self-efficacy in China, thereby strengthening their self-management capacity.

## Methods

### Data sources and search strategy

This study systematically searched the following databases: China National Knowledge Infrastructure (CNKI), Wanfang Data, VIP Database, SinoMed, PubMed, Web of Science, Embase, Scopus, and CINAHL. The search covered the period from the inception of each database to April 2025, aiming to identify studies related to the measurement properties of Chinese versions of self-efficacy assessment tools for rheumatoid arthritis (RA). The search strategy employed a combination of Medical Subject Headings (MeSH) and free-text terms, with appropriate adjustments tailored to the indexing characteristics of each database. Additionally, a snowballing approach was used to screen the reference lists of included articles to identify potentially relevant studies that may have been missed during the initial search. This study strictly adheres to the PRISMA statement [13] and COSMIN guidelines [12]. The search strategy was developed based on the validated search filter for measurement properties proposed by the COSMIN group [14]. The search strategy for this study is presented in Appendix 1. This study was registered with PROSPERO (registration number available upon request).

### Inclusion and exclusion criteria

#### Inclusion criteria

(1)The study population included patients with RA; (2) The objective of the study was the development, cross-cultural adaptation, or validation of a self-efficacy assessment tool for RA; (3) The study evaluated at least one measurement property of a self-efficacy assessment tool for RA. (4) The study was published in a peer-reviewed journal; (5) The publication language was English or Chinese.

#### Exclusion criteria

(1)The tool was designed to measure only a single dimension of self-efficacy (e.g., medication adherence, exercise self-efficacy); (2) Studies involving non-Chinese populations; (3) The study was a secondary research article (e.g., review, systematic review, conference abstract); (4) The full text of the article was not available.

### Study screening and data extraction

Two reviewers independently performed literature screening and data extraction based on the COSMIN guidelines. Any disagreements during the screening or extraction process were resolved through discussion; if consensus could not be reached, a third reviewer was consulted to resolve the issue. All three researchers involved in the screening process had completed an evidence-based nursing course and received systematic training on the latest COSMIN guidelines.

Literature management was conducted using EndNote version 21.4, and duplicates were removed through both automated and manual processes. During the initial screening phase, filters such as language and publication type were applied, and titles and abstracts were reviewed to preliminarily exclude studies that did not meet the inclusion criteria (e.g., irrelevant study population or topic). Full texts were then reviewed to determine final eligibility.

Extracted data included the first author, year of publication, study region, name of the assessment tool, sample size, number of dimensions and items in the scale, response options, test-retest interval, test-retest sample size, and questionnaire completion time, among other basic information.

### Quality appraisal

This study conducted a systematic quality appraisal of the measurement properties of PROMs based on the COSMIN framework [12]. According to the COSMIN guidelines, the recommended evaluation procedure involves three steps: first, the methodological quality of the included studies is assessed using the COSMIN Risk of Bias checklist to determine the risk of bias in psychometric studies [15]; second, the results of each measurement property are rated against the criteria for good measurement properties [12]; and finally, the quality of evidence is graded using a modified GRADE approach, based on which a recommendation level is provided [16].

#### Methodological quality appraisal

The methodological quality of content validity, internal structure, and other measurement properties was assessed using the COSMIN Risk of Bias checklist. The content validity domain included both the development process of the PROM and the evaluation of its content validity. The internal structure domain covered structural validity, internal consistency, and cross-cultural validity/measurement invariance. Other measurement properties assessed included reliability, measurement error, criterion validity, hypothesis testing for construct validity, and responsiveness. The checklist comprises 10 domains and 116 items, each rated as “very good,” “adequate,” “doubtful,” or “inadequate” according to predefined criteria, with “not applicable” used where appropriate. The rating of each domain followed the “worst score counts” principle, whereby the overall methodological quality of a domain was rated as “inadequate” if at least one item within the domain was scored as “inadequate” [15].

#### *Measurement property appraisal*

(1) **Content Validity**: Content validity refers to the extent to which the items of the tool adequately reflect the construct intended to be measured, and it is considered the most critical measurement property [17]. The COSMIN working group has established ten standards for evaluating content validity, including five for “relevance,” one for “comprehensiveness,” and four for “comprehensibility” [18]. In practice, the assessment of content validity should integrate the quality of the development process of PROM, the methodological quality of content validity studies, and the reviewer’s rating. Each of the three domains—relevance, comprehensiveness, and comprehensibility—is rated as “sufficient,” “insufficient,” or “indeterminate.” The overall content validity rating is then summarized as “sufficient,” “insufficient,” or “inconsistent.” Typically, an overall rating of “indeterminate” is not assigned, as reviewer opinions are generally available. According to COSMIN guidelines, content validity should be prioritized as the primary property to be assessed. If high-quality evidence demonstrates that a tool has “insufficient” content validity, further evaluation of additional measurement properties is not necessary, and the quality of evidence and recommendation can be determined directly. (2) **Other Measurement Properties**: Based on the criteria for good measurement properties [12], this study also evaluated the quality of structural validity, internal consistency, reliability, cross-cultural validity/measurement invariance, and hypothesis testing. Each property was rated as “sufficient,” “indeterminate,” or “insufficient.” The COSMIN guidelines recommend that structural validity be prioritized as the second most important measurement property, as the assessment of internal consistency depends on the quality of structural validity. For structural validity, if exploratory factor analysis (EFA) is conducted and the cumulative variance explained exceeds 50%, or if all factor loadings are above 0.40, the property is rated as “sufficient”; otherwise, it is rated as “insufficient” [19]. (3) **Hypothesis Testing**: Following COSMIN guidelines, the research team proposed the following hypotheses to assess construct validity: correlation coefficients between instruments measuring the same construct should exceed 0.50; correlations with instruments measuring related but distinct constructs should range between 0.30 and 0.50; and correlations with instruments assessing unrelated constructs should be less than 0.30.

### Evidence level synthesis and recommendations

#### *Quality synthesis*

Based on the evaluation results of the aforementioned measurement properties, two reviewers graded the quality of evidence for each property using a modified GRADE approach and formulated corresponding recommendations. For content validity, the quality of evidence was downgraded based on three factors: risk of bias, inconsistency, and indirectness. Given that the assessment relied on reviewers’ opinions, the overall quality of evidence for content validity was rated as “very low.” For other measurement properties, the evidence was downgraded based on four factors: risk of bias, inconsistency, imprecision, and indirectness. Initially, the quality of evidence for each measurement property was assumed to be “high” and was subsequently downgraded according to the aforementioned criteria. Final ratings were categorized as “high,” “moderate,” “low,” or “very low.”

#### *Recommendation*

Based on the summarized measurement property results and corresponding evidence quality, recommendations were made for each instrument as follows: Category “A”: The instrument demonstrates “sufficient” content validity at any level of evidence quality, and “sufficient” internal consistency supported by at least “low” quality evidence, which can be recommended for use. Category “B”: Instruments that do not meet the criteria for category “A” or “C” may be provisionally recommended, but further validation studies are needed to confirm their psychometric quality. Category “C”: The instrument has “insufficient” measurement properties supported by “high-quality” evidence, which should not be recommended for use.

## Results

### Literature search

**Fig. 1:**
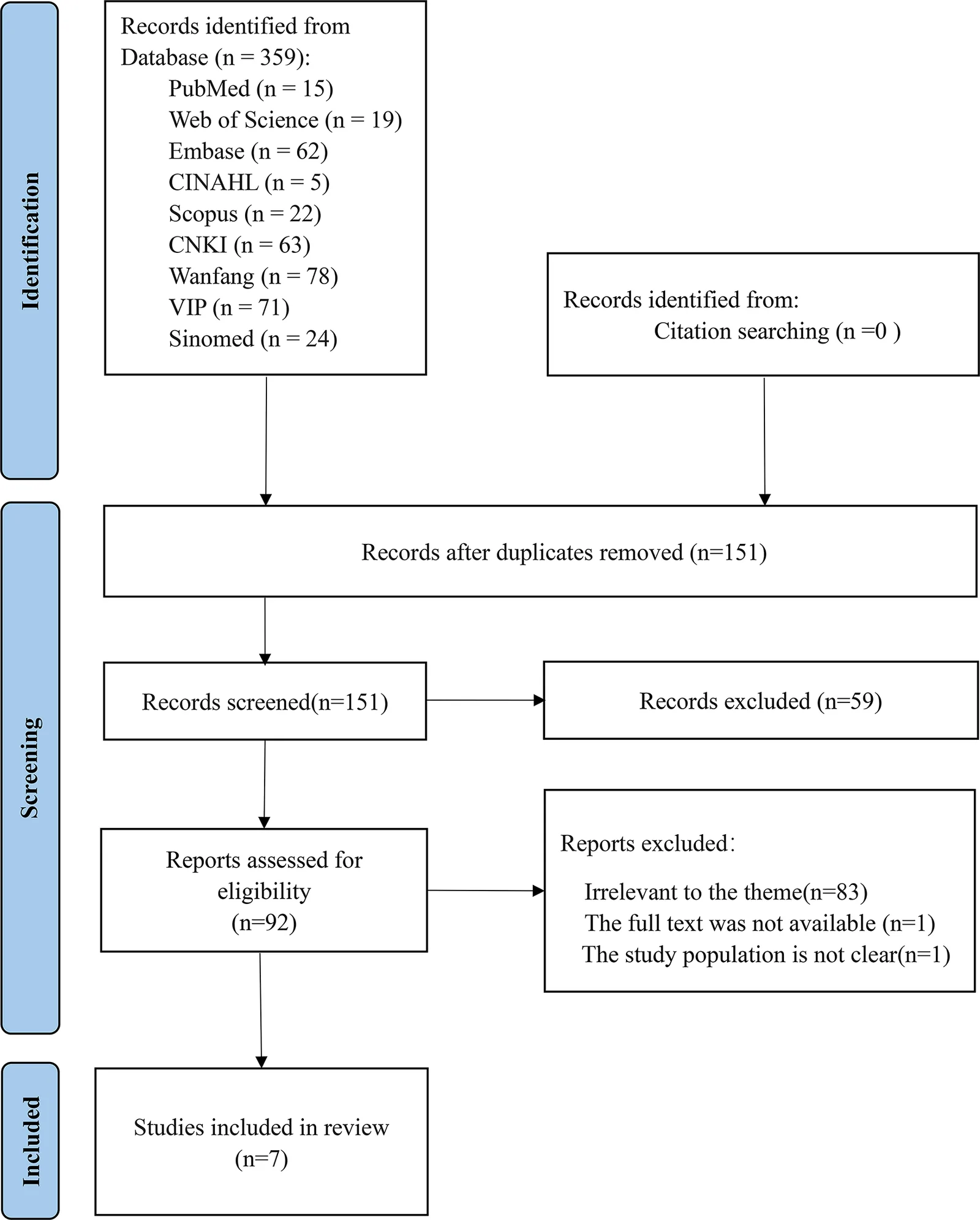
Literature screening process

A total of 359 records were identified through systematic searches of Chinese and English databases. After removing duplicates using both automated and manual methods, 151 records remained for initial screening. Following a stepwise screening process based on the predefined inclusion and exclusion criteria, 7 studies were ultimately included for analysis [20–26]. The literature selection process is illustrated in Fig. 1.

### Study description

All seven studies included in this review were cross-cultural adaptation and translation studies of Chinese versions of internationally developed self-efficacy assessment tools, covering seven instruments: ASES, ASES-8, JP-SES, and RASE. Among these, only one study included a partial sample of patients with conditions other than RA [20], while the remaining six studies focused exclusively on RA patients. In total, 36 psychometric evaluations were reported across the seven studies, including 10 for content validity, 7 for structural validity, 7 for internal consistency, 7 for reliability, and 5 for hypothesis testing of construct validity. Notably, three studies evaluated the Chinese version of the RASE, and two assessed the Chinese version of the JP-SES. During the translation and cross-cultural adaptation process, the included studies varied in their handling of item numbers and dimensional structures, resulting in inconsistencies that precluded pooled analysis. Consequently, each version of the Chinese-translated instruments was evaluated independently. The basic characteristics of the included studies are presented in Table 1.

**Table 1:** Basic characteristics of the included literature (*n* = 7)

first author	publication year	study location	Instrument name	Sample size	Gender female (% )	Age Range (Mean ± SD) Year	dimensions/items	Retest intervals/ Sample size	Response options	Time to complete(min)	Measurement Property Indicators
Tsai, T.Y. [20]	2020	China-Taiwan	C-ASES	258	192 (74.4)	57.1 ± 14.1	3/20	2w/96	10-point scale	/	**EFA**: cv = 59.78% **Cronbach’s α** = 0.82–0.91 **ICC** = 0.85–0.90, **Convergent validity** →ASES and TDQ: -0.53ཞ-0.69
Gao L [21]	2016	Tianjin	C-ASES-8	134	101(75.4)	28–80(58.5 ± 11.4)	1/8	1w/20	10-point scale	5	**EFA**: cv = 71.35% **Cronbach’s** α = 0.942, **ICC** = 0.98 **Convergent validity** →ASES-8 and SF-36 PF, FACIT-F: 0.561, 0.660, ASES-8 and pain-VAS, HADS-D, HADS-A,༚-0.487ཞ-0.656
Zhao, W.H. [22]	2019	Tianjin	C-JP-SES	105	84 (80.0%)	55 (median)	1/10	6month /101	4-point scale	20	**CFA**: RMSEA = 0.061, CFI = 0.99, **Cronbach’s** α = 0.922 **ICC** = 0.94 **Convergent validity** →JP-SES and PEPPI, DAS-28, HAQ: 0.326、-0.493、-0.64
Gu C F [23]	2020	Yancheng	C-JP-SES	200	148(74)	35–72(58.55 ± 13.9)	2/10	2w/60	5-point scale	10	**EFA**: cv = 49.81% **CFA**: CFI = 0.866 **Cronbach’s** α = 0.96, **ICC** = 0.84, **Convergent validity** →JP-SES and RASE:0.86
Li X W [24]	2019	Tianjin	C-RASE	101	91/10	25–86(57 ± 14)	9/25	2w/26	5-point scale	20	**EFA**: cv = 68.36% **Cronbach’s α** = 0.751, **Pearso***n* = 0.731
Sun Y [25]	2019	Tianjin	C-RASE	188	152(80.9%)	(21–91)61.4 ± 11.5	8/28	1w/30	5-point scale	5–12	**EFA**: cv = 68.55% **Cronbach’s** α = 0.660–0.901 **ICC**:0.819–0.984 **Convergent validity** →RASE and ASES-8: 0.292–0.549
Zhao S P [26]	2019	Sichuan	C-RASE	92	66(71.7%)	55.5 ± 10.6	7/27	2w/30	5-point scale	5–10	**EFA**: cv = 70.243% **Cronbach’s α** = 0.916 **ICC**: 0.719

Note: EFA =Exploratory Factor Analysis; CV = cumulative variance; ICC = Intraclass Correlation Coefficient; CFA = Confirmatory Factor Analysis; RMSEA =Root Mean Square Error of Approximation; CFI = Comparative Fit Index; “/” = not reported. PEPPI: perceived efficacy of patient physician interactions, DAS: Disease Activity Score, HAQ: Health Assessment Questionnaire, HADS: Hospital Anxiety and Depression Scale, VAS: Visual Analog Scale, SF-36 PF: physical functioning subscale of Short Form 36-Item Health, FACIT-F: Functional Assessment of Chronic Illness Therapy Fatigue, TDQ: Taiwanese Depression Questionnaire

### Methodological quality and measurement property assessment

None of the seven studies assessed cross-cultural validity/measurement invariance, measurement error, responsiveness, or criterion validity of the instruments. The measurement properties and methodological quality assessments of each instrument are summarized in Table 2.

#### *Content validity*

Following the COSMIN guidelines, this study systematically evaluated the content validity of the included instruments based on ten criteria: five for relevance, one for comprehensiveness, and four for comprehensibility [18]. All seven studies involved the cross-cultural adaptation of internationally developed instruments. As none reported the original development process of the instruments among RA, the risk of bias for instrument development was not assessed. Therefore, the subsequent evaluation of content validity was primarily based on the methodological quality of the content validity studies and/or the reviewer’s rating. Tsai et al. conducted a survey involving both rheumatology professionals and RA patients to evaluate the relevance, comprehensiveness, and comprehensibility of the instrument’s items [20]. As a result, the content validity of the C- ASES was rated as “sufficient.” However, due to the lack of information regarding the qualifications of the investigators and the methodological rigor of data analysis, the study’s methodological quality was rated as “doubtful.” The remaining six studies did not provide a comprehensive evaluation across all three domains of content validity (relevance, comprehensiveness, and comprehensibility). Four studies assessed patients’ comprehension of the items [21, 22, 24, 25], and this dimension was rated as “sufficient.” One study conducted a relevance survey with nursing professionals [23], and this domain was also rated as “sufficient.” Another study involved rheumatology professionals in the assessment of item relevance and comprehensiveness [26], both of which were rated as “sufficient.” However, as these studies did not meet the COSMIN-recommended sample size requirements, their methodological quality was uniformly rated as “doubtful.” For domains not reported, reviewer opinions were used for rating, and the methodological quality was downgraded to “inadequate.” Based on the literature [27–29], the research team proposed that a valid RA-specific self-efficacy instrument should include the following domains: (1) confidence in managing symptoms such as pain, fatigue, and stiffness; (2) confidence in addressing social limitations such as interpersonal relationships, leisure, and work; and (3) confidence in implementing disease management strategies including medication, physical activity, and sleep regulation. The C- JP-SES did not include items addressing symptom management [22, 23]; thus, the “comprehensiveness” domain was rated as “insufficient.” In contrast, the Chinese versions of the RASE and ASES-8 demonstrated greater comprehensiveness [21, 24–26], adequately capturing the self-efficacy constructs relevant to RA patients. The item wording was also clear and accessible, facilitating comprehension. Accordingly, the research team rated the relevance, comprehensiveness, and comprehensibility of these two instruments as “sufficient.”

#### Structural validity

All seven included studies evaluated the structural validity of the instruments using exploratory factor analysis (EFA) and/or confirmatory factor analysis (CFA). Three studies applied EFA [20, 21, 25], and their methodological quality was rated as “adequate.” In two studies, the sample size was less than five times the number of items [24, 26], resulting in a methodological quality rating of “inadequate.” Among the two studies that conducted CFA, one reported a comparative fit index (CFI) of 0.866 [23], which did not meet the COSMIN-recommended threshold of ≥ 0.95, and thus the structural validity was rated as “insufficient.” The other study reported a CFI value of 0.99 [22], meeting the COSMIN criteria, and the structural validity was rated as “sufficient.”

#### Internal consistency

All seven included studies assessed the internal consistency of the instruments. One study did not report Cronbach’s α coefficients for the subscales [26], and its methodological quality was rated as “inadequate.” Two studies only reported item-total correlations without providing Cronbach’s α coefficients [23, 24]; thus, their methodological quality was rated as “doubtful.” The remaining studies systematically reported Cronbach’s α coefficients for each subscale using appropriate statistical methods, and their methodological quality was rated as “very good.” Regarding the quality of internal consistency, three studies reported only the overall Cronbach’s α values for the overall scale [23, 24, 26], which were 0.96, 0.751, and 0.916, respectively. As subscale-specific coefficients were not provided, the internal consistency quality for these studies was rated as “indeterminate.” The results of one study [25] showed that Cronbach’s α coefficients for both subscales of the Chinese version of the RASE were below 0.7, leading to an internal consistency quality rating of “insufficient.” In contrast, three studies [20–22] reported Cronbach’s α coefficients exceeding 0.7 across all subscales, and the internal consistency quality was rated as “sufficient.”

#### Reliability

All seven included studies assessed the test–retest reliability of the instruments. According to the COSMIN guidelines, an optimal test–retest interval is approximately two weeks. In one study, the retest interval was six months [22], which was considered excessively long; thus, the methodological quality was rated as “inadequate.” In two studies, the retest interval was one week [21, 25]; however, the appropriateness of this interval was not justified, and the methodological quality was rated as “doubtful.” In addition, four studies did not explicitly report whether the patients’ health status remained stable or whether the measurement conditions were consistent between the two assessments [20, 23, 24, 26], leading to a “doubtful” rating for methodological quality. Regarding the quality of reliability, six studies reported intraclass correlation coefficient (ICC) values ranging from 0.719 to 0.984, all exceeding the acceptable threshold of 0.7 [20–23, 25, 26], indicating “sufficient” reliability quality. One study did not report ICC values [24], and the reliability quality was rated as “indeterminate.”

#### *Hypotheses testing for construct validity*

A total of five studies evaluated hypothesis testing for the construct validity of the instruments [20–23, 25]. The comparator instruments included the Taiwanese Depression Questionnaire (TDQ), the Hospital Anxiety and Depression Scale (HADS), the Functional Assessment of Chronic Illness Therapy-Fatigue (FACIT-F), the Short Form 36 Health Survey (SF-36) physical functioning subscale, the Laffrey Health Conception Scale (LHCS), the Perceived Efficacy in Patient–Physician Interactions scale (PEPPI-10), and the Disease Activity Score-28 (DAS-28). Regarding the methodological quality assessment, we strictly followed the COSMIN Risk of Bias checklist (Box 9a): if the comparator instrument had sufficient measurement properties in a population similar to the study population, it was rated as “very good”; if the comparator instrument was known to have sufficient measurement properties but it was uncertain whether they applied to the study population, it was rated as “adequate”; if only some information on measurement properties in any study population was available, it was rated as “doubtful”; if no information was available or there was evidence of insufficient measurement properties, it was rated as “inadequate.” Although the included studies stated that the comparator instruments had good reliability and validity, they did not provide evidence regarding the applicability of TDQ, HADS, FACIT-F, SF-36, LHCS, and PEPPI-10 to the rheumatoid arthritis (RA) patient population. Consequently, the methodological quality of three studies was rated as “adequate” [20–22]. Regarding the quality of hypothesis testing for construct validity, four studies [20–23] reported correlation coefficients between the target instruments and comparator instruments that were consistent with the hypothesized direction and magnitude, resulting in a quality rating of “sufficient.” However, in one study [25], the correlation coefficients between each subscale of the instrument and the comparator measures did not align with the hypothesized direction, and thus, the quality of hypothesis testing for construct validity was rated as “insufficient.”

**Table 2:** Methodological quality and measurement characteristics of the included tools (*n* = 7)

Instrument		content validity		structural validity	internal consistency	reliability	hypotheses testing
	relevance	comprehensiveness	comprehensibility				
C-ASES [20]	D/+	D/+	D/+	A/+	V/+	D/+	A/+
C-ASES-8 [21]	I/+	I/+	D/+	A/+	V/+	D/+	A/+
C-JP-SES [22]	I/+	I/-	D/+	V/+	V/+	I/+	A/+
C-JP-SES [23]	D/+	I/-	I/+	V/-	D/?	D/+	V/+
C-RASE [24]	I/+	I/+	D/+	I/+	D/?	D/?	/
C-RASE [25]	I/+	I/+	D/+	A/+	V/-	D/+	V/-
C-RASE [26]	D/+	D/+	I/+	I/+	I/?	D/+	/

Note: Methodological quality / Measurement Property; “V” = Very good, “A” = adequate, “D” = Doubtful, “I” = inadequate༛“+” = sufficient, “–” = insufficient, “?” = indeterminate༛“/”= not reported

**Table 3:** Summary of quality and recommendations for the included tools (*n* = 7)

Instrument	content validity		structural validity		Internal consistency		reliability		hypotheses testing		Recommendations
	Overall rating	Evidence quality	Overall rating	Evidence quality	Overall rating	Evidence quality	Overall rating	Evidence quality	Overall rating	Evidence quality	
C-ASES [20]	+	VL	+	L	+	VL	+	VL	+	L	B
C-ASES-8 [21]	+	VL	+	M	+	M	+	VL	+	M	A
C-JP-SES [22]	±	/	+	H	+	H	+	VL	+	M	B
C-JP-SES [23]	±	/	-	H	？	/	+	VL	+	H	C
C-RASE[24]	+	VL	+	VL	？	/	？	/	/	/	B
C-RASE [25]	+	VL	+	M	-	M	+	VL	+	H	B
C-RASE [26]	+	VL	+	VL	？	/	+	VL	/	/	B

Note: “+” = sufficient, “?”=indeterminate, “−” = insufficient, “±” = inconsistent “H” = high, “M” = moderate, “L” = low, “VL” =very low

“/”= NA

### Grading the quality of evidence and recommendations

The quality of evidence for each measurement property was evaluated in accordance with the COSMIN guidelines. Structural validity was used as the starting point for assessing the quality of evidence for internal consistency. The overall quality of evidence was then downgraded, where applicable, based on four factors: risk of bias, inconsistency, imprecision, and indirectness. Indirectness was identified in one study [20], where the study population did not exclusively consist of patients with rheumatoid arthritis. Consequently, all reported measurement properties for the Chinese version of the ASES in this study were downgraded by one level. the downgrading decisions for each instrument are summarized below.

- Chinese version of ASES [20]: Risk of bias: content validity (− 2), structural validity (− 1), reliability (− 2), hypothesis testing (− 1), Imprecision: reliability (− 1),Indirectness: all measurement properties (− 1)
- Chinese version of ASES-8 [21]: Risk of bias: content validity (− 3), structural validity (− 1), reliability (− 2), hypothesis testing (− 1),Imprecision: reliability (− 2)
- Chinese version of JP-SES [22]: Risk of bias: reliability (− 3), hypothesis testing (− 1)
- Chinese version of JP-SES [23]: Risk of bias: reliability (− 2), Imprecision: reliability (− 1)
- Chinese version of RASE [24]: Risk of bias: content validity (− 3), structural validity (− 3)
- Chinese version of RASE [25]: Risk of bias: content validity (− 3), structural validity (− 1), reliability (− 2),Imprecision: reliability (− 2)
- Chinese version of RASE [26]: Risk of bias: content validity (− 3), structural validity (− 3), reliability (− 2),Imprecision: reliability (− 2)

In several cases, certain measurement properties could not be rated due to “inconsistent” or “indeterminate” results. A comprehensive summary of the quality of evidence and corresponding recommendation levels for all included instruments is presented in Table 3.

According to the COSMIN guidelines on formulating recommendations, a tool is recommended as “A” if it meets the following two criteria: (1) content validity is rated as “sufficient” (regardless of the level of evidence quality, even if rated as “very low”); (2) internal consistency is rated as “sufficient” (with at least low-quality evidence). Based on these criteria, the Chinese version of the ASES-8 demonstrated “sufficient” content validity [21], with moderate-quality evidence supporting its “sufficient” internal consistency. This tool was categorized as “A” and is recommended for use. High-quality evidence indicated “insufficient” structural validity for the Chinese version of the JP-SES (two-factor structure) [23], classifying this tool as category “C” and not recommended for use. The remaining instruments did not meet the criteria for either category “A” or “C” and were categorized as “B”, meaning they may be temporarily recommended but require further validation.

## Discussion

This systematic review evaluated the measurement properties of Chinese versions of self-efficacy assessment tools for patients with RA. A total of seven self-efficacy instruments applicable to RA patients were included. Among these, the Chinese version of the Arthritis Self-Efficacy Scale-8 (ASES-8) met the criteria for category “A” and is recommended for use in relevant research and clinical practice. The evaluation results demonstrated that most measurement properties of the included instruments were satisfactory. However, certain studies exhibited methodological and reporting limitations. To ensure a systematic and rigorous evaluation process, this review strictly adhered to the COSMIN guidelines and conducted a standardized and comprehensive quality assessment of all relevant measurement properties.

### *Content validity*

Content validity refers to the extent to which the items of a RA self-efficacy scale adequately reflect the construct it is intended to measure [17]. The assessment of this property primarily includes three dimensions: relevance, comprehensiveness, and comprehensibility. These dimensions evaluate the scale’s coverage of the target construct during both the development and finalized stages, as well as the degree to which patients can understand the items [18]. Specifically, relevance and comprehensiveness should be assessed through qualitative or quantitative studies involving both patients and professionals, whereas comprehensibility primarily focuses on patients’ understanding and requires only patient-based investigations. In the included studies, no systematic or rigorous content validity studies were conducted during the cross-cultural adaptation of the instruments. For instance, Tsai et al. [20] only invited four rheumatology experts to assess the comprehensiveness and relevance of the scale items, without incorporating feedback from RA patients. Although Gu et al. [23] proposed a pre-survey involving 60 patients, the specific content and methodology of the survey were not clearly described; therefore, the corresponding data were excluded from the present analysis. None of the seven studies met the sample size requirements recommended by the COSMIN guidelines, nor did they clearly report whether the interviewers possessed relevant expertise or had undergone standardized training. As a result, there was a high risk of methodological bias in the study of content validity.

### Structural validity

All included instruments in this review were based on reflective models. According to the COSMIN guidelines, structural validity should be systematically evaluated using both EFA and CFA. During the cross-cultural adaptation process, item modifications or deletions are often necessary; therefore, it is recommended to first conduct EFA to explore the underlying structure, followed by CFA to assess the model’s goodness-of-fit. For example, in the study by Gu et al. [23], during the translation and adaptation of the JP-SES scale, principal component analysis extracted two factors, with cumulative variances explained of 49.81%. CFA was then conducted, yielding a comparative fit index (CFI) of 0.866, which did not meet the recommended psychometric standards (i.e., cumulative variances explained > 50%, CFI > 0.95). The potential reason for these suboptimal results may be the insufficient sample size, which limited the accurate verification of the instrument’s underlying structure. Furthermore, the JP-SES scale may be more suited to a unidimensional structure, as supported by other research findings [22], where CFA results showed a CFI of 0.99, indicating a good model fit. Although two other studies reported EFA results meeting the quality standards for structural validity [24, 26], their evidence quality should be downgraded by three levels according to the COSMIN guidelines due to sample sizes being smaller than five times the number of items. Therefore, during the translation and cross-cultural adaptation of instruments, it is crucial to integrate feedback from both experts and the target population, while also conducting structural validity analyses with adequate sample sizes to improve measurement precision and ensure psychometric robustness.

### Internal consistency

Internal consistency refers to the degree of consistency among items within a scale, reflecting the measurement precision of the items for the same underlying construct. Before evaluating internal consistency, the factor structure of the scale should be identified through factor analysis. Therefore, compared with reporting only the overall Cronbach’s α coefficient for the entire scale, reporting the internal consistency coefficients for each dimension provides more informative and meaningful evidence [12]. In this review, although the Chinese versions of the JP-SES and RASE scales demonstrated multidimensional structures [23, 26], the relevant studies only reported overall Cronbach’s α coefficients, without providing separate internal consistency indicators for each dimension. As a result, the internal consistency quality of these instruments was rated as “uncertain.” To more comprehensively assess the reliability of these scales, future studies are recommended to clearly determine the factor structure and report Cronbach’s α coefficients for each dimension separately. This approach would allow for a more accurate reflection of item consistency within each dimension, thereby enhancing the scientific rigor and comparability of the evaluation results.

### Reliability

All included studies in this review adopted test-retest reliability as the method for assessing reliability, which involves administering the same measurement tool to the same participants at two different time points to evaluate the consistency of the results. The fundamental assumption of test-retest reliability is that the underlying construct being measured remains stable during the interval between measurements. Therefore, reliability studies should establish an appropriate time interval, ensure consistent measurement conditions, and confirm that the participants’ health status remains unchanged during the interval. If the interval is too long, the construct being measured may undergo actual changes; if it is too short, the results may be influenced by recall bias from the first measurement. According to the COSMIN guidelines, a retest interval of approximately two weeks is recommended for PROMs. However, in one study [23], the retest interval was extended to six months without providing a reasonable justification for this time frame, which constitutes a significant methodological flaw. Moreover, for continuous variables, the intra-class correlation coefficient (ICC) is considered a more appropriate indicator of test-retest reliability than Pearson or Spearman correlation coefficients, as it accounts for both within-subject random error and systematic bias caused by time, making it more suitable for reliability assessment. In summary, future studies involving instrument development or cross-cultural adaptation are recommended to select appropriate statistical methods for reliability assessment based on the type of data, establish reasonable measurement intervals, and provide clear justifications for these decisions. In addition, effective communication with patients should be emphasized during the implementation of reliability studies to ensure their understanding and cooperation, thereby enhancing the accuracy and scientific rigor of the collected data.

### Research on the measurement properties of chinese versions of rheumatoid arthritis self-efficacy instruments remains incomplete

The four self-efficacy instruments included in this review were all adapted from original foreign-language scales through translation into Chinese, yet significant limitations exist in terms of cross-cultural adaptation. Firstly, none of the included studies assessed cross-cultural validity or measurement invariance (MI) of the instruments. In future cross-cultural adaptation studies, it is recommended to conduct measurement invariance (MI) or differential item functioning (DIF) analyses across different subgroups (e.g., by country, gender, or cultural background) to assess whether the instruments measure the same underlying constructs consistently across populations [17]. When conducting cross-cultural validity analyses, it is essential to control for other relevant variables apart from the grouping variable to enhance the interpretability and scientific rigor of the results. Furthermore, none of the included studies analyzed or reported measurement error or responsiveness. Measurement error refers to deviations in the measurement results caused by systematic and random errors, which do not reflect true changes in the construct being measured. According to the COSMIN guidelines, appropriate statistical indicators should be selected to evaluate measurement error based on the type of data [30]. For continuous data, recommended indicators include the standard error of measurement (SEM), limits of agreement (LOA), and smallest detectable change (SDC), while for categorical or ordinal data, percentage agreement is recommended. Responsiveness, which reflects an instrument’s sensitivity to changes in the construct over time, is a critical attribute for evaluating the dynamic validity of a scale. Similar to hypothesis testing for construct validity, responsiveness should be evaluated by comparing the instrument’s results with those of a comparator tool based on predefined hypotheses. However, none of the included studies assessed responsiveness. With regard to criterion validity, which examines the agreement between scale scores and a “gold standard,” no such analyses were conducted in the included studies, likely due to the absence of an established gold standard for assessing self-efficacy in RA. According to the COSMIN guidelines, in the absence of a clearly defined gold standard, the original full-length version of the scale may serve as a reference standard for evaluating shortened versions [30]. However, in studies [23, 25], the so-called “criterion validity” assessments were essentially conducted using hypothesis testing for construct validity, which does not align with the COSMIN definition of criterion validity. In summary, although existing studies have explored content validity, structural validity, and internal consistency to some extent, systematic evaluations of key measurement properties such as cross-cultural validity/measurement invariance, measurement error, criterion validity, and responsiveness are still lacking. Future research on the development and validation of RA self-efficacy instruments should comprehensively address these measurement properties to enhance the scientific rigor, applicability, and cross-cultural generalizability of the scales.

## Limitations

(1) Due to resource limitations, this study only included published literature available in the databases and did not incorporate grey literature, which may have led to a certain degree of publication bias.

(2) Although the C-ASES-8 met the COSMIN criteria for recommendation, the methodological quality of some measurement properties was rated as “doubtful” or “inadequate,” and the reporting of measurement properties in the included study was incomplete. Consequently, the strength of the evidence supporting the recommendation remains limited, and the conclusions of this systematic review should be interpreted with caution.

(3) Although the COSMIN Risk of Bias Checklist provides structured and transparent guidance—particularly when independent raters reach consensus—its application still requires subjective interpretation, which may introduce variability in the assessment process. As noted by the COSMIN developers, the checklist “is not a cookbook,” and its use inherently involves expertise and subjective judgment [31]. This subjectivity represents a potential source of variability inherent to any COSMIN-based evaluation. Future methodological efforts could focus on refining specific items to reduce ambiguity and further enhance the consistency of assessments across reviews.

## Conclusion

This study systematically evaluated the measurement properties of Chinese versions of self-efficacy assessment tools for patients with RA following the COSMIN guidelines. The results demonstrated that the C-ASES-8 provides a relatively comprehensive assessment of patients’ self-efficacy in symptom management, social functioning limitations, and disease management, meeting the recommended criteria for use. Currently, there is a lack of self-developed self-efficacy assessment tools specifically designed for RA patients in China. It is recommended that future tool development strictly follow the COSMIN Risk of Bias Checklist, adopt scientifically rigorous research designs, and conduct systematic and comprehensive evaluations and reporting of all relevant psychometric properties to develop high-quality assessment tools suitable for Chinese RA patients.

## Supplementary Information

Below is the link to the electronic supplementary material.

Supplementary Material 1

## Data Availability

The author confirms that all data generated or analyzed during this study are included in this published article.
