# Supplementary material for: Measurement properties of Chinese-version assessment tools of self-efficacy in patients with rheumatoid arthritis: a COSMIN-based systematic review
Source: J Patient Rep Outcomes. 2026 Jul 7;10:176. doi: 10.1186/s41687-026-01135-9 (PMC13627544; doi:10.1186/s41687-026-01135-9)
Supplement: Supplementary file 1 [file 41687_2026_1135_MOESM1_ESM.docx]

**Search Strategy**

**PUBMED**

(((((Arthrit*[Title/Abstract]) OR (Arthritis[MeSH Terms])) AND (("Efficacy, Self"[Title/Abstract] OR "Self-efficacy"[Title/Abstract] OR "Self efficacy"[Title/Abstract]) OR (Self Efficacy[MeSH Terms]))) AND ((Tool*[Title/Abstract] OR scale*[Title/Abstract] OR questionnaire*[Title/Abstract] OR instrument*[Title/Abstract]) OR (Surveys and Questionnaires[MeSH Terms]))) AND ((Reliab*[Title/Abstract] OR Valid*[Title/Abstract] OR develop*[Title/Abstract] OR test*[Title/Abstract] OR cross-cultural[Title/Abstract] OR psychometr*[Title/Abstract] OR Transcultur*[Title/Abstract] OR reproducib*[Title/Abstract] OR clinimetr*[Title/Abstract] OR clinometr*[Title/Abstract]) OR (Psychometrics[MeSH Terms]))) AND (Chin*[Title/Abstract])

**WOS**

#1Arthrit*[topic]

#2“Efficacy, Self” or “Self-efficacy” or “Self efficacy”

#3Tool* or scale* or questionnaire* or instrument*

#4Reliab* or Valid* or develop* or test* or cross-cultural or psychometr* or Transcultur* or reproducib* or clinimetr* or clinometr*

#5Chin*

**search query**：#1 AND #2 AND #3 AND #4 #5

**SCOPUS**

#1Arthrit*[ Article title,Abstract or keywords]

#2‘Efficacy, Self’ or ‘Self-efficacy’ or ‘Self efficacy’ [ Article title,Abstract or keywords]

#3Tool* or scale* or questionnaire* or instrument*[ Article title,Abstract or keywords]

#4Reliab* or Valid* or develop* or test* or cross-cultural or psychometr* or Transcultur* or reproducib* or clinimetr* or clinometr* [ Article title,Abstract or keywords]

#5 Chin*

**search query**：#1 AND #2 AND #3 AND #4 #5

**EMBASE**

**#1arthrit***:ti,ab,kw OR **'arthritis'**/exp

**#2'efficacy, self'**:ti,ab,kw OR **'self-efficacy'**:ti,ab,kw OR **'self efficacy'**:ti,ab,kw OR **'self concept'**/exp

#3tool*:ti,ab,kw OR scale*:ti,ab,kw OR questionnaire*:ti,ab,kw OR instrument*:ti,ab,kw OR 'questionnaire'/exp

**#4reliab***:ti,ab,kw OR **valid***:ti,ab,kw OR **develop***:ti,ab,kw OR **test***:ti,ab,kw OR **'cross-cultural'**:ti,ab,kw OR **psychometr***:ti,ab,kw OR **transcultur***:ti,ab,kw OR **reproducib***:ti,ab,kw OR **clinimetr***:ti,ab,kw OR **clinometr***:ti,ab,kw OR **'psychometry'**/exp

**#5chin***:ti,ab,kw

**search query**：#1 AND #2 AND #3 AND #4 #5

**CINAHL**

AB (Arthrit*) AND AB ("Efficacy, Self" or "Self-efficacy" or "Self efficacy") AND AB (Tool* or scale* or questionnaire* or instrument*) AND AB (Reliab* or Valid* or develop* or test* or cross-cultural or psychometr* or Transcultur* or reproducib* or clinimetr* or clinometr*) AND AB (Chin*)

**中国知网（CNKI）**

#1关节炎[篇关摘]

#2自我效能[篇关摘]

#3问卷 + 量表 + 工具 + 评估 + 评价[篇关摘]

#4编制 + 研制 + 检验 + 信度 + 效度 + 汉化 + 跨文化 + 测量属性 + 调试 [篇关摘]

**search query**：#1 AND #2 AND #3 AND #4

**万方医学（wangfang）**

#1关节炎[摘要]

#2自我效能[摘要]

#3问卷 or 量表 or 工具 or 评估 or 评价[摘要]

#4编制 or 研制 or 检验 or 信度 or 效度 or 汉化 or 跨文化 or 测量属性 or 调试 [摘要]

**search query**：#1 AND #2 AND #3 AND #4

**中国生物医学文献数据库（sinomed）**

"关节炎"[常用字段:智能] AND "自我效能"[常用字段:智能] AND( "问卷"[常用字段:智能] OR "量表"[常用字段:智能] OR "工具"[常用字段:智能] OR "评估"[常用字段:智能] OR "评价"[常用字段:智能]) AND( "编制"[常用字段:智能] OR "研制"[常用字段:智能] OR "检验"[常用字段:智能] OR "信度"[常用字段:智能] OR "效度"[常用字段:智能] OR "汉化"[常用字段:智能] OR "跨文化"[常用字段:智能] OR "测量属性"[常用字段:智能] OR "调试"[常用字段:智能])

**维普资讯（VIP）**

#1关节炎[摘要]

#2自我效能[摘要]

#3问卷 or 量表 or 工具 or 评估 or 评价[摘要]

#4编制 or 研制 or 检验 or 信度 or 效度 or 汉化 or 跨文化 or 测量属性 or 调试 [摘要]

**search query**：#1 AND #2 AND #3 AND #4
